# Optical coherence tomography angiography measured area of retinal neovascularization is predictive of treatment response and progression of disease in patients with proliferative diabetic retinopathy

**DOI:** 10.1186/s40942-020-00249-6

**Published:** 2020-11-04

**Authors:** Anna Stage Vergmann, Kristian Tølbøl Sørensen, Thomas Lee Torp, Ryo Kawasaki, Tien Wong, Tunde Peto, Jakob Grauslund

**Affiliations:** 1 Department of Ophthalmology, Odense University Hospital, Kloevervaenget 5, indgang 132, 5000 Odense C, Denmark; 2 Research Unit of Ophthalmology, Department of Clinical Research, Faculty of Health Science, University of Southern Denmark, Odense, Denmark; 3 Steno Diabetes Center Odense, Odense, Denmark; 4 OPEN, Open Patient Data Explorative Network, Odense University Hospital, Odense, Denmark; 5 Department of Vision Informatics, Osaka University School of Medicine, Osaka, Japan; 6 Singapore National Eye Centre, Singapore, Singapore; 7 Office of Clinical Sciences, Duke-NUS Medical School, National University of Singapore, Singapore, Singapore; 8 School of Medicine, Dentistry and Biomedical Sciences, Queen’s University, Belfast, Northern Ireland UK; 9 Visual Computing Lab, The Alexandra Institute, Aarhus, Denmark

**Keywords:** Optical coherence tomography angiography, Proliferative diabetic retinopathy, Fluorescein angiography, Diabetic retinopathy

## Abstract

**Background:**

The purpose of this study was to evaluate the area of retinal neovascularization in patients with treatment-naïve proliferative diabetic retinopathy (PDR) as measured by optical coherence tomography angiography (OCT-A) as a marker of subsequent treatment response after panretinal photocoagulation (PRP), and to examine if this area correlated with area of retinal neovascularization as measured by fluorescein angiography (FA).

**Methods:**

En face OCT-A scans (4.5 × 4.5 mm) of neovascularizations were obtained at baseline (BL) before PRP and at month (M) 3 and M6 after treatment. Progression of PDR were defined as lesion growth (assessed by ophthalmoscopy and wide-field fundus photo) or increasing leakage by Optos ultra-widefield FA, and patients were divided into two groups; progression or non-progression. Mann–Whitney U test and Wilcoxon signed-rank test were used to analyse differences between groups and between time points. Areas of retinal neovascularizations (OCT-A and FA) were calculated by algorithms developed in Python (version 3.6.8, The Python Software Foundation, USA).

**Results:**

Of 21 eyes included, 14 had progression of disease. Median OCT-A area did not differ between the two groups (progression vs. non-progression) at BL (76.40 ± 162.03 vs. 72.62 ± 94.15, p = 0.43) but were statistically significantly larger in the progression group at M6 (276.69 ± 168.78 vs. 61.30 ± 70.90, p = 0.025). Median FA area did not differ in the progression vs. the non-progression group at BL (111.42 ± 143.08 vs. 60.80 ± 54.83, p = 0.05) or at M6 (200.12 ± 91.81 vs. 123.86 ± 162.16, p = 0.62). Intraclass correlation between area by OCT-A and FA was −5.99 (95% CI: −35.28–0.993), p = 0.71.

**Conclusions:**

In this study of patients with treatment-naïve PDR, we showed that increasing area of retinal neovascularizations measured by OCT-A at M6 indicated progression of disease after PRP treatment. Our results suggest that area by OCT-A reflects disease activity and that it can be used as an indicator to monitor the progression of PDR over time, and to evaluate treatment response six months after PRP.

*Trial registration* https://clinicaltrials.gov (identifier: NCT03113006). Registered April 13, 2017.

## Background

Diabetic retinopathy (DR) is a major cause of vision impairment in working age adults, with the most severe stage, proliferative diabetic retinopathy (PDR) accounting for a large proportion of blindness [1, 2]. The Early Treatment Diabetic Retinopathy Study [3] demonstrated that fluorescein angiography (FA) is effective to determine disease activity in PDR, guiding prognosis and amount of panretinal photocoagulation (PRP) that physicians need to administer. However, FA is an invasive [4] and time-consuming procedure, and area of leakage for retinal neovascularization (NV), one of the indicators of severity, is difficult to evaluate objectively and over time [5]. Optical coherence tomography angiography (OCT-A) is a relatively new modality that detects the movement of red blood cells in retinal vessels [6]. It allows cross-sectional non-invasive assessment of retinal NV without the need for injection of dye. OCT-A is widely used in the diagnostics of choroidal NV [7, 8] and some research have been done into the field of the macular foveal avascular zone (FAZ) [9, 10] that has found FAZ to be larger in patients with diabetes and in patients with DR. Furthermore, in a previous study, it has been found that the flow increases in the macula following PRP, suggesting that OCT-A may also allow prognosis in patients severe DR [11]. Less is explored in the field of OCT-A as a diagnostic or prognostic tool in regards to PDR, but some studies have found valuable benefits of the procedure in describing the morphology of retinal NVs [12] and to evaluate preretinal NVs in PDR [13]. If OCT-A can assist or possibly even replace FA in assessment and monitoring of PDR, this would be a clinically useful tool that can save both time and unnecessary use of intravenous dye.

Therefore, the purpose of this study was to evaluate the area of retinal NV in patients with treatment-naïve PDR as measured by OCT-A as a possible marker of subsequent treatment response after PRP, and to examine if the OCT-A measurements correlated with area of retinal NV as measured by FA.

## Methods

The study was designed as a six-month 1:1 prospective randomized controlled trial (RCT) and included 53 eyes of 47 patients with treatment-naïve PDR. Patients were randomized to either 1) standard PRP with Navilas® (n = 27) or 2) individualized PRP with Navilas® (n = 26). The study was conducted at Odense University Hospital, Odense, Denmark, and the patients were included between June 1st, 2017 to February 1st, 2019. Randomization were performed by Research Electronic Data Capture database under Open Patient data Explorative Network. To ensure the same degree of ischemic disease, the two groups were balanced in relation to the number of retinal quadrants with retinal NVs. Patients were blinded to what treatment they received, and inclusion criteria were as following; diabetes mellitus (type 1 and 2), newly diagnosed, untreated PDR in one eye (possibility of inclusion of both eyes if bilateral PDR where each eye would be randomized in each treatment group). Exclusion criteria were DMO in the affected eye (central subfields thickness < 300 μm), age < 18 years, pregnancy, and/or blurry optic media that could prevent PRP. Treatment efficacy (progression of PDR) and side-effects (visual field, dark adaptation and retinal quality of life) were defined as main outcomes of the trial. Given that the main outcomes did not differ between patients with progression and patients without progression, eyes of both groups were pooled for the present study [14]. Patients were divided into two groups; progression and non-progression of PDR after treatment.

### Visual acuity and clinical examinations

Demographic information, a full medical history, height and weight (BMI) were collected at baseline (BL). At BL, month 3 (M3) and month 6 (M6) follow up the following were measured: best corrected visual acuity (BCVA) using the Early Treatment Diabetic Retinopathy Study (ETDRS) chart, intraocular pressure, brachial arterial blood pressure (Omron 705CP, Hoofdrop, The Netherlands) and venous blood samples of Haemoglobin A_1c_ (HbA1c), triglycerides, and cholesterol (low-density lipoprotein and high-density lipoprotein) were drawn and spectral domain (SD) optical coherence tomography (OCT) (Topcon, Tokyo, Japan) was performed. The patients underwent a standard ophthalmic examination including slit lamp examination performed in mydriasis with tropicamide 10 mg/mL (Mydriacyl) and phenylephedrine 10% (Metaoxedrin) and wide-field fundus photo and FA were performed at both BL, M3 and M6.

### Optical coherence tomography angiography and fluorescein angiography

**Fig. 1:**
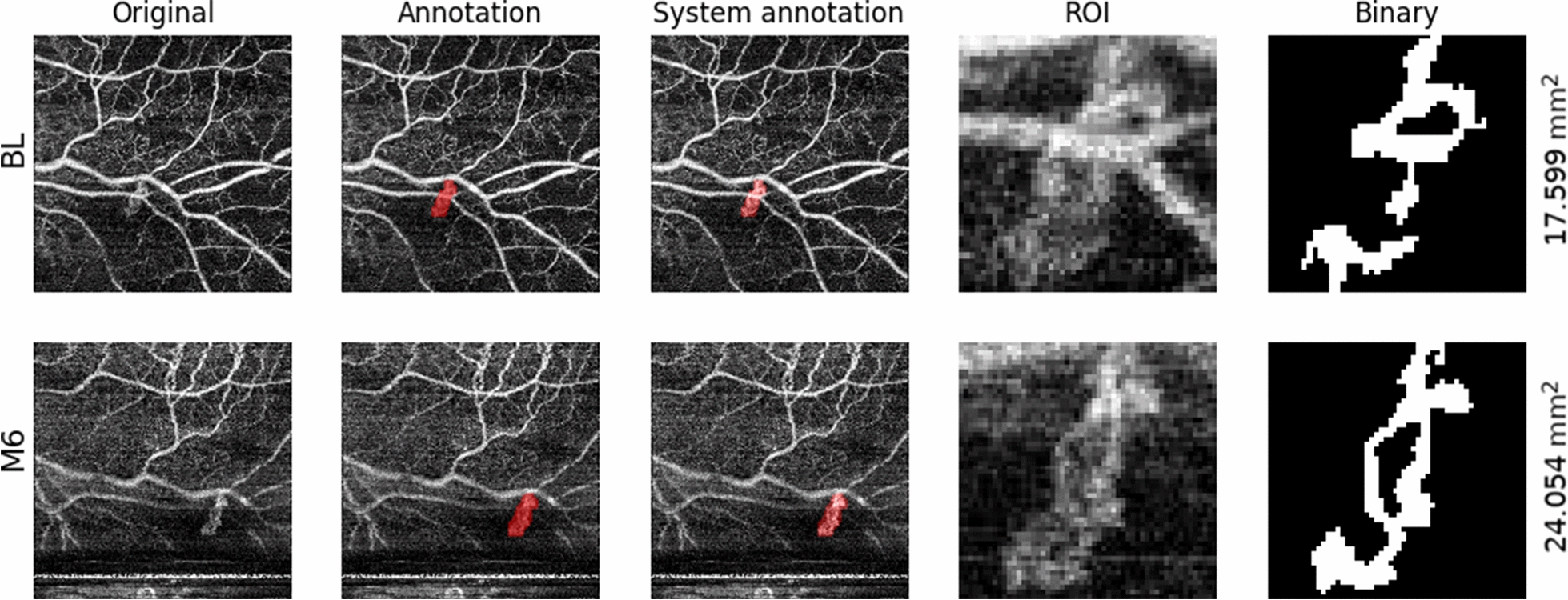
Optical coherence tomography angiography measured area of neovascularization

En face OCT-A scans (4.5 × 4.5 mm, DRI OCT Triton, swept source OCT, Topcon) of retinal NVs were obtained at BL, before PRP, and at M6. This OCT uses long wavelength scanning light (1,050 nm) and provides 100,000 A scans per second [15]. The scan was placed over the area(s) of the retina with retinal NVs which were detected by fluorescein angiography and indirect ophthalmoscopy. OCT-A could not be obtained in all eyes because of the placement of the retinal NV (too far in the periphery) or cooperation difficulties of the patients. Images were exported from ImageNet6 (Topcon, Tokyo, Japan) and the ‘Layer Modify’ tool was utilized to cover the area of the retinal NV, making sure everything was captured even. The pixels of the exported pictures were 320 × 320. To ensure continuity between scans across different timepoints (BL, M6), the same retinal area and layer were chosen for analysis. In one patient where we obtained two scans of two separate retinal NVs, the areas were calculated separately. Areas of retinal neovascularizations (OCT-A and FA) were calculated by algorithms developed in Python (version 3.6.8, The Python Software Foundation, USA). Area of NVs by OCT-A was determined as illustrated in Fig. 1. On the original grayscale image (first column), the region of interest (ROI) was indicated by author ASV manually masking potentially relevant pixels in a color (second column). A Python-algorithm was developed by author KTS to semi-quantitatively establish area of retinal NVs, as described in the Additional file 1 and summarized here: First, by comparing the annotated and original images, the annotated pixel mask was deduced (overlaid in the third column). The ROI was isolated by cropping the original image to a square centered around the annotation (fourth column). Finally, the grayscale image was reduced to a binary representation (fifth column) using adaptive thresholding, and the area of white pixels inside the mask was reported. Area of leakage by FA was measured by author ASV masking areas of interest. A Python-algorithm developed by author KTS was then used to count the number of pixels. Pixels were then converted into mm^2^ by measuring the diameter of the optic disc in pixels of each FA image and use a conversion-factor (known average size of optic disc (1.74 mm for Caucasians [16]). It should be noted that the instrument magnification between acquisitions was similar but not constant and that optic disc size can vary from patient to patient, rendering the pixel-to-micron conversion factor leading to some measurement uncertainty in the reported values.

### Panretinal photocoagulation

Standard PRP was localized to all four quadrants. Individualized PRP was localized only to the affected quadrant(s) [14]. Navilas® was used for treatment which is a 532-nm laser with integrated image-assistance and navigation. It tracks eye-movements and offer both single- and multi-spot laser treatment. Treatment was carried out at baseline (BL) and supplemented at M3 and / or M6 if needed. The treatment was performed in two sessions, with one week in between treatments, at BL in both groups. The patients were given a local anaesthetic (oxybuprocaine hydrochloride 0.4%) prior to treatment. A Mainster 165 PRP contact lens was used. The laser spot size was set to 390 μm and pulse duration 30 ms. Power was set to 280 milliwatt and increased or decreased until a clear white indication was present on the retina. Indications for additional treatment were progression of PDR as evidenced by lesion growth (assessed by ophthalmoscopy and wide-field fundus photo) or increasing leakage by wide-field Optos ultra-widefield (Optos, Dunfermline, United Kingdom) FA at M3 or M6 follow up.

### Ethical considerations

All patients participated on the basis of informed and written consent (publication included) and were informed that they at any time could withdraw from the study. The study was conducted in accordance with the Helsinki Declaration II and in accordance with good clinical practice. The project was approved by the Research Ethics Committee of Southern Denmark (Project-ID: S-20160168) and by The Danish Data Protection Agency. The full trial protocol can be found at https://clinicaltrials.gov (identifier: NCT03113006, https://clinicaltrials.gov/ct2/show/NCT03113006?term=NCT03113006&draw=2&rank=1), as the project was registered online prior to initiation.

### Statistics

Data are presented as median with interquartile range (IQR). Sample size calculations was performed from the primary endpoints in the main study[14]. Mann–Whitney U test and Wilcoxon signed-rank test were used to analyse differences between groups and between time points. Intraclass correlation was calculated for area of retinal neovascularizations measured by OCT-A and FA. Statistical analysis was performed using STATA Intercooled version 16 (StataCorp LLC, College Station, TX, USA).

## Results

Twenty-one OCT-A scans were obtained for 21 eyes at BL and 16 OCT-A scans for 15 eyes at M6. Patients characteristics can be seen in Table 1. The progression vs. non-progression groups did not differ in median age, sex, race, diabetes type, median body mass index, median diabetes duration, median BCVA, median systolic blood pressure, median diastolic blood pressure, or in median HbA1c. Of 21 eyes included, 14 had progression of disease.

**Table 1:** Baseline characteristics

Baseline characteristics	Progression	Non-progression	P-value
No. of eyes (n)			
21	14	7	
Age (years)	43.5 ± 28.5	61.0 ± 22.0	0.08
Sex, male (%)	75.0%	54.0%	0.33
Ethnicity, caucasian (%)	100.0%	100.0%	1.00
Body mass index (kg/m^2^)	26.1 ± 4.3	30.4 ± 6.9	0.31
Diabetes, type 1 (%)	75.0%	54.0%	0.05
Diabetes duration (years)	22.0 ± 9.0	17.0 ± 7.0	0.12
Best corrected visual acuity (ETDRS)	85.0 ± 5.0	80.0 ± 9.0	0.24
Systolic blood pressure (mmHg)	130.0 ± 45.0	144.0 ± 22.0	0.06
Diastolic blood pressure (mmHg)	79.0 ± 20.0	87.0 ± 12.0	0.44
HbA1c (mmol/mol)	70.0 ± 38.0	70.0 ± 7.0	0.59

Baseline characteristics of patients included in the study in the Progression and Non-progression groups. Data are presented as median with interquartile range or percentage

Median OCT-A area did not differ between the two groups (progression vs. non-progression) at BL (p = 0.43) but were statistically significantly larger in the progression group at M6 (p = 0.025). Median FA area did not differ in the progression vs. the non-progression group at BL (p = 0.05) or at M6 (p = 0.62). Results can be seen in Table 2.

**Table 2:** Area of retinal neovascularizations by optical coherence tomography angiography and fluorescein angiography

	BL		M6	
Progression (OCT-A area)	n = 14	76.40 ± 162.03	n = 6	276.69 ± 168.78
Non-progression (OCT-A area)	n = 7	72.62 ± 94.15	n = 10	61.30 ± 70.90
P-value	0.32		0.025*	
Progression (FA area)	n = 14	111.42 ± 143.08	n = 6	200.12 ± 91.81
Non-progression (FA area)	n = 7	60.80 ± 54.83	n = 10	123.86 ± 162.16
P-value	0.05		0.62	

Area of neovascularizations (mm^2^) by optical coherence tomography angiography (OCT-A) and fluorescein angiography (FA) divided into patients that progressed in their disease (progression) and patients that did not progress in their disease (non-progression) at follow up. Data are presented as median with interquartile range.

*BL* baseline, *M6* month 6 follow up

*Statistically significant

**Fig. 2:**
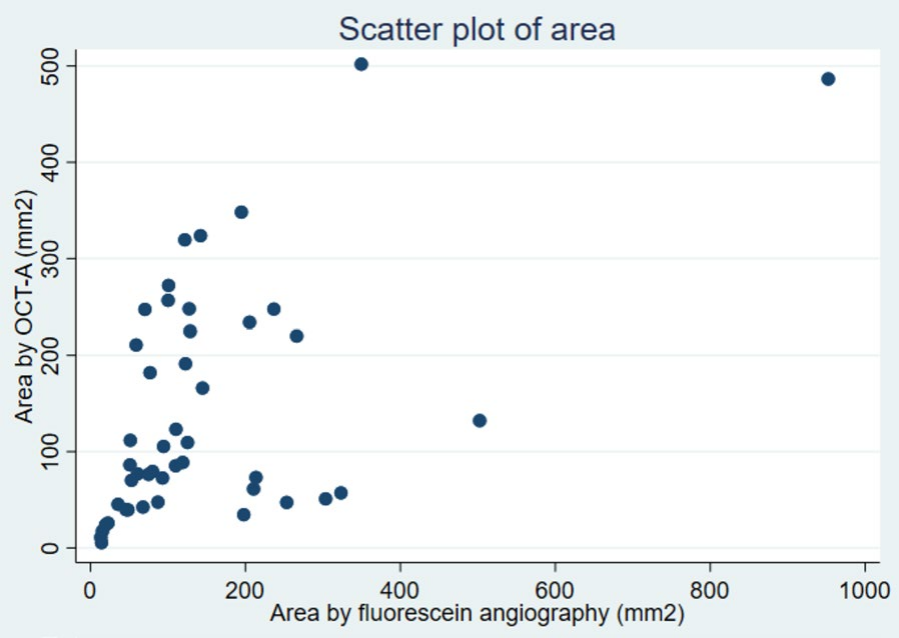
Scatter plot between area (mm2) measured by optical coherence tomography angiography (OCT-A) and fluorescein angiography

Intraclass correlation between area by OCT-A and FA was −5.99 (95% CI: −35.28;0.993), p = 0.71. A scatter plot representing the correlation of area measured by OCT-A and FA can be seen in Fig. 2. This scatter plot shows a trend towards OCT-A measuring areas larger than FA.

## Discussion

In this study of patients with treatment-naïve PDR, we demonstrated that increasing area of retinal NV measured by OCT-A was associated with progression of disease six months after treatment with PRP. Our results suggest that retinal NV area measured by OCT-A reflects disease activity and that it can be used as a tool to monitor the development of PDR over time, and furthermore to evaluate treatment response six months after PRP. However, OCT-A is not able to replace FA, but it can act as a good supplemental tool in the monitoring of the disease.

We investigated area of retinal NVs by OCT-A as an assessor of disease progression in patients with PDR treated with PRP. When diagnosing and monitoring PDR, FA is a commonly used tool to evaluate the disease activity. However, it is a time-consuming and invasive examination. Moreover, there is a subjective component to the evaluation of leakage because of the difficulty in quantifying the retinal NV due to relatively fast dye leakage and staining, and adverse events of injection of intravenous fluorescein have been described [4]. OCT-A is on the contrary non-invasive and an objective imaging modality of the different layers of the retina. We found that the area of retinal NVs measured by OCT-A were able to distinguish between progression and non-progression of PDR 6 months after initial treatment, with statistically significant larger areas in patients with progression in whom we were able to obtain OCT-A scans from. The pathophysiological mechanism behind PDR is growth of retinal NVs and this can be seen with progression of the disease. FA can still show signs of leakage from areas where there are no activity reflecting functional damage of the microvasculature (e.g., a loss of barrier function), where OCT-A only detects vessels with movement of red blood cells [6], hence areas with active flow of relatively severe condition. This, together with our findings, makes OCT-A a very useful tool to detect disease activity in PDR.

Even though OCT-A is a promising tool, it possesses some limitations which should be taken into consideration. Artefacts has been reported to be a limiting factor of the technique [17]. There are also variations in between the different software making it impossible to compare scans between machines from different developers. Additionally, the field of view is fairly narrow which together with cooperation issues from the patients was the cause of exclusion of 32 eyes from our RCT in this OCT-A sub study. Even though wide-field OCT-A has become available [18] it still lacks the ability to give a wide overview to screen the retina for retinal NVs, as for example Optos wide-field FA is capable of. Because of this, we do not believe that OCT-A can replace FA but is a good supplement in monitoring the disease after initial FA to identify all retinal NVs together with indirect ophthalmoscopy. In the future, wide-field OCT-A can hopefully be extended to a larger view of the retina and be the only tool necessary to diagnose and monitor retinal NVs in PDR.

A strength to our study was the automated measurements of the area of retinal NVs making the method objective compared to previous studies on this field [18, 19]. The method was deterministic, making sure the algorithm always output the same area if area by the same picture is measured more than once. Furthermore, the algorithm did not have knowledge about which patients progressed in their disease and was therefore not biased when assessing the area. In subjective assessment, even of OCT-A, the knowledge of the patient’s disease status can maybe lead the examiner in a certain direction. If the algorithm can be developed for easy use, it has potential to be an objective clinically helpful tool. However, the area of interest to analyse was noted by an individual which gives the method a semi-quantitative quality, but the actual measurements of area was solely done by the algorithm. Another strength to our study was the prospective design, making it possible to evaluate retinal NVs over time.

One limitation to this study was the limited numbers of images available because of difficulties with the equipment and the narrow field of view (4.5 × 4.5 mm) of the available scanner. This made it very difficult to reach retinal NVs that where further than 3 OD away from the centre of the retina. Because of this, we were not able to obtain scans from the periphery of the retina, this resulting in the low number of scans. Poor cooperation from the patients also played a part in this. The patients needed to focus on a point for a certain amount of time to obtain the scan in the right position and not all patients were capable of this. Furthermore, the Triton Topcon OCT is designed to perform OCT-A primarily in the macula that is flat, and this makes it difficult to get an image in good quality when moving away from the centre of the retina. The limited number of patients and the potential uncertainty in the golden standard of progression/non-progression should also be taken into consideration. In this study, we did not find a correlation between area by OCT-A and FA [20]. This is in conflict with a previous study on this topic that found a good correlation of area measured by the two methods [21]. When measuring area of retinal NVs by OCT-A the measurements are only done of the relevant diseased vessels. When measuring area of leakage by FA this is much more diffuse with leakage not always representing the actual size of the NVs. This was also demonstrated in our scatter plot (Fig. 2), where there was a trend towards a larger area measured by OCT-A (except for a few outliers) than measured by FA. This could maybe indicate that FA actually underestimate the severity of the disease. Furthermore, the analysis of area of retinal NVs by FA in our study should be interpreted with caution. The export of the pictures did not make it possible to get the exact same size and resolution of every picture and the optic disc conversion factor we used could render some uncertainties.

## Conclusions

In this study of patients with treatment-naïve PDR, we showed that increasing retinal NV area measured by OCT-A indicated progression of disease after treatment with PRP. Our results suggest that retinal NV area measured by OCT-A reflects disease activity and that it can be used as an additional tool to monitor the development of PDR over time, and to evaluate treatment response six months after PRP. In this study, OCT-A were better at visualizing progression of disease at follow up, compared to FA. Nonetheless, there are still some issues with OCT-A which should be addressed before implementing it as a sole diagnostic and monitoring tool of PDR.

## Supplementary information

**Additional file 1.** Supplementary information.

## Data Availability

Data is stored in a Research Electronic Data Capture web-based secure database under Open Explorative Network, Odense. The datasets generated and analysed during the current study are not publicly available due to patient confidentiality but are available from the corresponding author on reasonable request.

## Abbreviations

PDR: Proliferative diabetic retinopathy
PRP: Panretinal photocoagulation
FA: Flourescein angiography
OCT-A: Optical coherence tomography angiography
NV: Neovascularization
BL: Baseline
M3: Month 3 follow up
M6: Month 6 follow up
