# Supplementary material for: Optical coherence tomography angiography measured area of retinal neovascularization is predictive of treatment response and progression of disease in patients with proliferative diabetic retinopathy
Source: Int J Retina Vitreous. 2020 Nov 4;6:49. doi: 10.1186/s40942-020-00249-6 (PMC7640645; doi:10.1186/s40942-020-00249-6)
Supplement: Supplementary file 1 — Additional file 1. Supplementary information. [file 40942_2020_249_MOESM1_ESM.pdf]

Supplementary information

The data analysis algorithm measures the number of bright pixels within a region. This section explains what constitutes a "bright" pixel, and how the coordinates inside the region of interest is defined. For the analysis, OpenCV 4.1.0.25 was used in Python 3.6.8.

SI: Mask extraction

The contour is manually defined by the user drawing a colored contour around the region of interest. In order to identify which pixels to include in the analysis, pixels located inside this contour are first identified. Figure 1 in the article illustrates this process:

1) Original data with hand-drawn contour defining the region of interest. This is the input image. As the raw data is grayscale, anything colored can be assumed to be the contour, and the next three steps ensure that the particular color does not matter:
2) Comparison between the red and green color channels. The variation is ca. 0.5e6.
3) Comparison between the red and blue color channels. The variation is ca. 0.7e6, indicating that the blue channel holds most information.
4) Comparison between the green and blue color channels. The variation is ca. 0.2e6.
5) The difference between the blue channel and the original image blue channel of the annotation image is subjected to a thresholding, such that any pixel value smaller than 32 is set to zero.
6) Binarization ensures that all pixels are either 0 or 1.
7) Morphological closing using a 5-pixel square structuring element. This ensures that the contour is a closed shape, even if there is a few pixels of gap somewhere in the contour (common when using a thin contour line).
8) A black border is added to avoid erroneous closing in the next step:
9) Morphological filling is used to fill the connected component. Note that in this example, without the border added in the previous step, the entire region to the right of the contour would be filled as well, as the contour touches the edge in two points.
10) The extracted region of interest is visualized by overlaying red pixels inside contour.

SI: Area measurement

In order to determine whether a pixel should be included in the measured area, a human would compare the intensity and shape of the structure to the background. Although humans are highly adept at recognizing patterns despite significant noise, reproducibility is likely to be poor from sample to sample and person to person. In order to increase reproducibility and objectivity, a quantitative method was developed using computer vision analysis.

It should be noted that the manual determination of the region of interest is a major variable affecting quality of analysis, and this aspect of the analysis will be somewhat subjective. However, subsequent steps always follow the same process with identical parameters. The steps of the analysis are explained in figure 1 of the article:

1) Input region of interest, the part of the image covered by the manual contour, from which a mask was extracted as explained above.
2) The image is inverted.

3) The inverted image is median blurred using a 3x3 structuring element in order to remove high-frequency noise.
4) Adaptive thresholding is applied, calculating a varying threshold in blocks of 15 pixels. A bias of 13 is subtracted from the mean.
5) The image is inverted again.
6) Connected components analysis is used to remove blobs of area less than 30.
7) Finally, the mask is applied to ignore structures outside the region of interest.
